# Between the Devil and the Deep Blue Sea: Non-Coding RNAs Associated with Transmissible Cancers in Tasmanian Devil, Domestic Dog and Bivalves

**DOI:** 10.3390/ncrna7040072

**Published:** 2021-11-10

**Authors:** Nicholas C. Lister, Ashley M. Milton, Benjamin J. Hanrahan, Paul D. Waters

**Affiliations:** School of Biotechnology and Biomedical Sciences, Faculty of Science, UNSW Sydney, Sydney, NSW 2052, Australia; a.milton@unsw.edu.au (A.M.M.); b.hanrahan@unsw.edu.au (B.J.H.); p.waters@unsw.edu.au (P.D.W.)

**Keywords:** transmissible, CRISPR/Cas, cancer, ncRNAs, Tasmanian devil, bivalve, domestic dog

## Abstract

Currently there are nine known examples of transmissible cancers in nature. They have been observed in domestic dog, Tasmanian devil, and six bivalve species. These tumours can overcome host immune defences and spread to other members of the same species. Non-coding RNAs (ncRNAs) are known to play roles in tumorigenesis and immune system evasion. Despite their potential importance in transmissible cancers, there have been no studies on ncRNA function in this context to date. Here, we present possible applications of the CRISPR/Cas system to study the RNA biology of transmissible cancers. Specifically, we explore how ncRNAs may play a role in the immortality and immune evasion ability of these tumours.

## 1. Transmissible Cancers

Cancers are usually self-limiting in that tumour cells perish with the host and are not transmitted to others in the population [1]. Traditionally, cancer was only believed to be a transmissible disease under exceptional circumstances [2]. These circumstances have been referred to as a ‘perfect storm’, and include several cancer cell characteristics (e.g., phenotypic plasticity), as well as host factors (e.g., low host genetic diversity) [2,3]. To become transmissible, a cancer must have a route to access, infect and colonise additional hosts [3].

In rare cases, a cancer can be naturally transmitted from one human to another, e.g., during pregnancy [4]. The immune systems of both mother and foetus tolerate foreign antigens during pregnancy, allowing human to human transmission to occur [4]. There have been numerous cases in which a mother has developed cancer while pregnant, and the cancer has ‘metastasised’ to the developing child [4]. Cancers can also be naturally transmitted to a human host from a different species. In one unique case, a human HIV patient developed a tapeworm (*Hymenolepis nana*) cancer, which was presumably due to immune deficiencies and pre-existing tapeworm colonisation [5].

Additionally, some cancers have been artificially transmitted between individuals in medical settings (through transplants and surgical accidents) and laboratory experiments (e.g., transplantation of tumours between animals) [3,6,7]. A repeated example of artificially transmitted cancer in humans is from donor to recipient in organ transplants where the immunosuppressed state of the recipient permits a foreign tumour to proliferate [8]. Donor screening was improved in 1997, which lowered the incidence of transmitted cancer from 30% to 0.05% [9,10].

**Figure 1:**
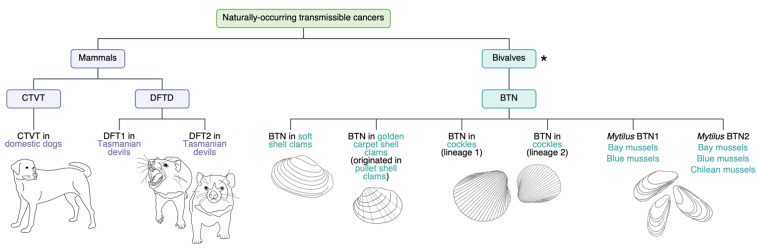
Currently known independently evolved transmissible cancer lineages observed in wild populations. In mammals, canine transmissible venereal tumour (CTVT) and two lineages of devil facial tumour disease (DFTD) have been described. In bivalves, there are six distinct examples of bivalve transmissible neoplasia (BTN). * This figure represents currently confirmed BTN lineages. The number of bivalve species affected by BTN is likely to be underestimated [11].

The above cases involve cancer transmission between two individuals only, i.e., from mother to child, organ donor to recipient, or tapeworm to a human patient. These cancers do not spread through the population. This contrasts at least nine naturally occurring transmissible cancers in dogs, Tasmanian devils and bivalves that infect multiple individuals in the population (Figure 1). In fact, transmissible cancers may be more common in nature than represented in the literature [11].

### 1.1. Canine Transmissible Venereal Tumour (CTVT)

The first naturally occurring transmissible cancer discovered was canine transmissible venereal tumour (CTVT), having been observed in domestic dogs around the world over the last two hundred years [12,13]. It has been experimentally transmitted to wild canids such as wolves, coyotes and red foxes, but there are no confirmed incidents of CTVT occurring in wild populations [14]. CTVT is believed to have originated in a dog related to Alaskan Malamutes approximately 4000–8500 years ago [13,15], making it the most prolonged proliferating mammalian cell line [16]. CTVT is sexually transmitted and generally non-fatal to the host as it regresses after three to nine months [16]. Although widespread, its non-lethality results in minimal effect on dog populations and reproduction, creating a stable coexistence of host and ‘pathogen’ that has developed over thousands of years.

The genome of CTVT has undergone large scale structural alterations, as well as gene specific changes in expression. CTVT has a diploid number of 2n = 57–59, in contrast to the domestic dog’s 2n = 76 [17]. This reduced diploid number is likely the result of fusion events between small chromosomes, leading to 16–18 bi-armed chromosomes [17]. Specific marker chromosomes are present, which vary by geographic region [16]. A change characteristic of CTVT is the insertion of a LINE1 upstream of the c-*myc* oncogene [18]. Increased expression of c-*myc* in CTVT may be a result of this insertion [18,19].

Further changes in gene expression have enabled CTVT to persist as a transmissible cancer. Telomerase is upregulated, which presumably maintains telomere length [2,20]. CTVT achieves downregulation of dog leukocyte antigen genes DLA-I and DLA-II (the canine equivalent of MHC-I and -II) via secretion of transforming growth factor β (TGF-β) [2,16]. Their under-expression aids CTVT in evading the host immune system [2]. However, dogs are usually immune to re-infection after the tumour regresses, indicating that the current immune escape adaptations of CTVT do not permit unchecked growth [16].

### 1.2. Devil Facial Tumour Disease (DFTD)

In contrast to the relatively innocuous and ancient CTVT is the more recently discovered devil facial tumour disease (DFTD). DFTD was first observed in wild Tasmanian devils in 1996 (DFT1) [21]. DFTD is a transmissible facial tumour that is spread primarily by biting behaviour during mating and feeding. It causes death in approximately six months [22,23]. In 2014, a second DFTD emerged in wild devils (DFT2) [24]. Both of these transmissible tumours are derived from neuroectodermal tissues, but cytogenetic and transcriptomic evidence show that they originated independently in different individuals [25,26]. DFT1 originated in a female devil; it has two rearranged X chromosomes and no Y chromosome [27,28]. DFT2 contains a Y chromosome, so originated in a male individual [24]. DFTD has had a severe impact on its host population. Local populations declined more than 80% in the first 5 years after DFT1 discovery, and there was an estimated average decline of 77% across all DFTD-affected populations to 2018 [29,30].

Both DFT1 and DFT2 have substantial karyotypic differences compared to the normal Tasmanian devil karyotype. DFT1 has extensive rearrangement of chromosome 1 and the X [28], and four characteristic marker chromosomes [22]. In DFT2, one copy of chromosome 6 has been inserted into chromosome 2 to form a larger chromosome [26]. Additional material is also present on chromosomes 1 and 4 and there is a deletion involving chromosome 5 [24]. At a smaller scale, the alteration of particular genes may contribute to DFTD’s success. For example, there is a homozygous deletion of the gene TP73 in DFT2 [26]. TP73 plays a role in activating apoptosis [31], which might contribute to uncontrolled proliferation of DFT2.

As in CTVT, telomerase is upregulated in DFT1 [32]. This upregulation is the result of increased expression of the catalytic subunit of telomerase: telomerase reverse transcriptase (TERT) [32].

An important feature of both DFTD tumours is altered major histocompatibility complex (MHC) expression. The MHC is a family of genes in the mammalian adaptive immune system involved in self/non-self-recognition by T cells [11]. MHC class I (MHC-I) molecules are not expressed on the surface of DFT1 cells [33]. This contrasts DFT2, in which MHC-I genes are expressed. However, it has been suggested that this expression in DFT2 could become downregulated over time [34,35]. MHC downregulation in both DFTDs would hinder the host’s ability to identify foreign cells. Although MHC mRNA is produced, it was shown that epigenetic downregulation of antigen-processing genes, rather than physical mutation, caused the lack of MHC-I expression on the cell surface of DFT1 [33].

Despite DFTD adaptations for immune evasion, some Tasmanian devils are evolving an immune response to DFT1. Remarkably, there has been evidence of selection for genes involved in cancer or immune function over only ~4–6 generations [36]. Initially, DFT1 was thought to be 100% fatal. This is no longer the case, with multiple individuals from West Pencil Pine, Tasmania found to survive the disease or exhibit tumour regression [37]. Interestingly, a 2016 study showed that the IgM/IgG (Immunoglobulin M/Immunoglobulin G) expression ratio in the host had a statistically significant effect on DFT1 status. Higher IgM expression compared to IgG was associated with lower presence of DFT1 in Tasmanian devils [38]. The strong selective pressure imposed by DFTD is driving a rapid evolutionary response. Perhaps one day Tasmanian devil populations will be less affected by DFT1 and DFT2, like dog populations are by CTVT.

### 1.3. Bivalve Transmissible Neoplasia (BTN)

Disseminated neoplasia, a leukaemia-like cancer, has been observed for decades in various bivalve species, including oysters [39,40,41,42]. Since 2015, several species were confirmed to have independently evolved transmissible tumours (bivalve transmissible neoplasia—BTN) [43]. These include: soft shell clams (*Mya arenaria*), bay mussels (*Mytilus trossulus*), cockles (*Cerastoderma edule*), golden carpet shell clams (*Polititapes aureus*), Chilean mussels (*Mytilus chilensis*) and blue mussels (*Mytilus edulis*) [44,45] (Figure 1).

There are also multiple examples of cross-species cancer transmission. The tumour observed in golden carpet shell clams is believed to have originated from pullet shell clams, despite low levels of neoplasia in pullet shell clams [46]. Mytilus BTN1 and Mytilus BTN2 are separate tumours that developed in bay mussels [47]. These two tumours are observed in both bay mussels and blue mussels, whereas only Mytilus BTN2 is observed in Chilean mussels [45,47]. Another two distinct BTN lineages arose independently in cockles [44]. The emergence of multiple BTNs in these bivalves mirrors the independent rise of two DFTD tumours.

It is probable that many more BTNs exist and are yet to be identified, with modelling showing that transmissible cancers are likely to be more common in nature than reported [11]. As BTN continues to be studied, more bivalve species are being added to the list of known hosts. Mediterranean mussels (*Mytilus galloprovincialis*) are also potentially BTN-affected. Disseminated neoplasia has been widely observed in this species, but its transmissibility is yet to be confirmed [45].

In BTN and bivalve non-transmissible disseminated neoplasia, invasive cells are found in the circulatory fluid [2]. These invasive cells can sequester TP53 protein in the cytoplasm, express novel surface antigens and lose phagocytic and apoptotic abilities, leading to displacement, compression, and host cell death in the circulatory system [2,48,49]. The sequestering and inactivation of TP53 is noteworthy, as p53 is a tumour suppressor gene [49].

Unlike CTVT and DFTD, direct contact is not required for transmission of BTN. Bivalves are filter feeders, so BTN cells can be shed into seawater and dispersed on currents to access new hosts [2,43]. It was shown that haemocytes from a soft shell clam with leukaemia could exist in seawater for at least six hours, and were tolerant to environmental changes, with only low levels of cell death occurring [50].

Disseminated neoplasia and BTN can cause mass mortalities in bivalve populations. High mortality has been previously associated with disseminated neoplasia in wild oysters in Australia, Spain and Croatia [51,52]. As BTNs have the potential to cause bivalve population declines [2], they pose a threat to aquaculture should a tumour infect farms. Importantly, effects of climate change such as ocean warming will likely facilitate outbreaks of disseminated neoplasia [53]. Therefore, it is possible that BTN will become more of a threat into the future. The potential detriments for bivalve aquaculture are clear, which is a global industry that produced 14.65 million tons—worth approximately $24 billion (USD)—in 2015. Of this, China had by far the largest production with 12.4 million tons alone, worth $18.5 billion (USD). The rest of Asia produced the next highest amount with 1.1 million tons, followed by Europe with 0.6 million tons [54].

## 2. Non-Coding RNAs

The number of protein coding genes in an organism was once thought to be directly related to an organism’s complexity. Conversely, regions considered to be without function or transcriptional activity were designated as ‘junk DNA’ [55,56]. This notion changed when the genomes of organisms with lower complexity, such as mouse [57], nematode worm [58] and chicken [59] were discovered to have a similar number of protein coding genes as humans. RNA was long thought of as only an information intermediary between DNA and protein. However, the many functions that RNA performs within the cell are now appreciated, as previously unknown capabilities of RNA are uncovered [60]. New genome-wide technologies such as chromatin immunoprecipitation sequencing (ChIP-seq) [61] and RNA sequencing (RNA-seq) [62,63,64] have improved capabilities to identify and understand the roles of RNA. This has resulted in the emergence of new hypotheses about how genes are regulated.

Cases of structural non-coding RNAs (ncRNAs) carrying out basic functions were recorded in the 1950s. These ncRNAs included ribosomal RNAs (rRNAs) [65] and transfer RNAs (tRNAs) [66], both involved in translation. The 1990s introduced the concept of ncRNAs with varying roles and functions with the discoveries of XIST [67,68], which plays an important role in X chromosome inactivation, and H19 [69,70], which undergoes paternal imprinting.

While originally considered rare, ncRNAs have a large role in gene regulation [71,72]. Only a small part (1.2%) of the human genome encodes for proteins, whereas approximately 70% is transcribed into RNA [71,72,73]. Most of the human genome is transcribed into ncRNAs with complex overlapping patterns [60,71,74,75]. RNA sequencing technologies has led to identification of tens of thousands of ncRNAs, however many have no ascribed function because mechanistic studies of individual ncRNAs are much more involved [71,72,76].

NcRNAs are characterised as either short ncRNAs (<200 nt) or lncRNAs (>200 nt). MicroRNAs (miRNAs) are the most well studied of the short ncRNAs, though there are also short-antisense RNAs (sasRNAs), piwi interacting RNAs (piRNAs), short interfering RNAs (siRNAs), small nucleolar RNAs (snoRNAs) and transcription initiating RNAs (tiRNAs). There is also a variety of lncRNAs, such as antisense RNAs (asRNAs) [72], transcribed ultraconserved regions (T-UCRs) [77], enhancer RNAs [78], transcribed pseudogenes [79] and large intergenic ncRNAs (lincRNAs) [61].

Non-polyadenylated lncRNAs have been identified, although by and large most lncRNAs are polyadenylated [80,81]. LncRNAs are involved in a number of biological processes. Antisense lncRNAs have regulatory roles in metabolism, cell cycle and stem cell pluripotency [80,82,83]; one example is epigenetic regulation of HIV viral transcription [84]. Gene expression can be regulated either transcriptionally or post-transcriptionally, and lncRNAs can be *cis* or *trans* acting [82,85]. Gene regulation is altered through interaction of lncRNAs with transcription factors and/or chromatin remodelling proteins [83,86,87].

An antisense RNA is complimentary to it partner sense-expressed transcript, normally a protein coding mRNA. Antisense RNAs overlap promoters, UTRs (both 5′ and 3′), introns and exons. *Tsix*, antisense to *XIST* at the X-inactivation centre [88], and the parent of origin silencing (genomic imprinting) of *KCNQ1* [89] and *IGF2R* [90] were some of the first examples of asRNA gene regulation. AsRNA transcription occurs throughout the human genome, which has been observed through cap analysis of gene expression (CAGE) [72] and strand specific analysis of expressed sequence tags [91].

It is now established that asRNAs play vital roles in numerous cellular processes, such as RNA stability [92,93], epigenetic remodelling [94], translation [95], altered mRNA splicing [96] and imprinting [88]. A variety of cancer-associated genes (such as *p53* [93], *p15* [97], and *p21* [95]) are regulated by asRNAs. Upregulation of the lncRNA HOTAIR is linked to poor prognosis and increased metastasis in a number of different tumours in humans, including breast, lung, gastric and pancreatic cancers [98]. HOTAIR is known to interact with members of the PRC2 complex, specifically SUZ12 and EZH2, which are involved in methylation of H3K27 [99]. Inhibition of HOTAIR led to downregulation of the PI3K/AKT pathway and *MRP1*, as well as increased sensitivity to the drug imatinib, a tyrosine kinase inhibitor [100]. PTEN is a tumour suppressor gene that also regulates the PI3K/AKT pathway and has a known lncRNA regulator itself, *PTENP1* [101,102]. Increased expression of the lncRNA MALAT1 correlates with poor prognosis in lung cancer, whereas reduced MALAT1 expression leads to reduced lung cancer cell motility, an indicator of lowered metastatic ability [98]. These are just some examples of the known roles for ncRNAs in cancer.

NcRNAs are vital for gene regulation in many biological processes and have a clear role in cancer [92,93,94,95,96,97,103]. Much work has identified and annotated ncRNAs in humans and other model species like mouse, but less is known for non-traditional model species. There is a large reduction of non-coding genes and pseudogenes identified in Tasmanian devil and dog compared to humans (Ensembl database). Popular opinion might attribute this to the perceived increased complexity of humans and the requirement for large numbers of ncRNAs to subtly regulate gene expression. However, much of the disparity likely results from fewer genomic studies in devils and dogs.

## 3. CRISPR/Cas

The CRISPR/Cas system is capable of introducing specific genetic changes to cultured cells or animal models of interest. It is derived from a prokaryotic immune defence in which foreign viral DNA is recognised and cleaved by the Cas protein [104]. The CRISPR/Cas complex is comprised of an RNA-guided DNA endonuclease (Cas9) and a single guide RNA (sgRNA). The sgRNA itself is made up of two components: a CRISPR RNA (crRNA) component, which directs the complex by homologous base pairing with the target sequence [105], and a trans-activating crRNA (tracrRNA) component, which is involved in the maturation of crRNA as well as binding it to the Cas protein [106]. A genomic target sequence must be followed by a protospacer adjacent motif (PAM), which is any base followed by two guanine bases or an adenine and guanine (i.e., NGG or NAG) [107]. Expressing Cas9 and a sgRNA results in precision cutting of the target, causing double stranded breaks. Non-homologous end joining (NHEJ) and homology directed repair (HDR) can be utilised to insert known sequence into the cleavage site or cause deletions [108], which can induce deleterious mutations that inactivate a target gene to model disease outcomes.

A catalytically inactivated version of the Cas complex (dCas9), which does not cleave the underlying DNA sequence, can be fused to proteins that either activate or repress target genes. CRISPRi fuses dCas9 to transcriptional repressors such as the KRAB domain [109,110], whereas CRISPRa fuses dCas9 to transcriptional activators [111]. In traditional CRISPR applications a plasmid containing cassettes for the Cas9 protein and sgRNA is introduced to target cells, which will then be expressed for a short time. These cells are then screened for the desired genomic changes. Conversely, CRISPRa and CRISPRi technologies rely on expression of the dCas9 and guide RNA for extended periods over which the effects can be measured. dCas-fusion proteins have also been developed that can affect epigenetic modifications. For example, DNA methylation at specific CpGs can be increased in targeted areas of the genome using DNA methyltransferase dCas9 fusion proteins [112].

The versatility of the CRISPR has led to its utilisation in all aspects of medical and biological research, especially cancer research. The occurrence and proliferation of cancer is characterised by many genetic changes, usually in oncogenes or tumour suppressor genes [113]. Homologous recombination (HR) and NHEJ have been used to create mouse models and cell lines with specific mutations. This was combined with site-specific recombinases such as Cre and flippase to generate conditional alleles for many genes associated with cancer [114]. However, these approaches suffer from low efficiency of gene targeting and large time requirements for generation of mouse models. CRISPR has opened an array of techniques that include rapid modelling of genetic events related to cancer, as well as much faster generation of mouse models, and somatic genetic engineering ex vivo and in vivo [115].

While CRISPR is a versatile tool for modelling mutation and controlling protein coding gene expression, there are challenges when applied to lncRNAs. LncRNAs are present throughout the genome in both inter- and intra-genic regions [76]. If lncRNAs of interest are located within a protein coding gene, CRISPR mediated alteration of the expression (or alteration of the lncRNA sequence) could affect protein coding genes on the same or opposite strands. Despite this, CRISPR has been used to successfully alter the action of lncRNAs, by introducing a termination signal or RNA destabilising elements immediately downstream of the lncRNA TSS [116,117].

CRISPRa was used to overexpress the lncRNA Interferon Gamma Antisense 1 (IFNG-AS1) in a study of inflammatory bowel disease, causing a 20-fold increase, showing the efficacy of the use of CRISPRa on lncRNAs [118]. In contrast, CRISPRi was used in vivo in *Drosophila* to supress the expression of the lncRNAs *rox1* and *rox2* [119]. This demonstrated the effective use of CRISPRi, in vivo, to repress expression of lncRNAs by 50-fold.

CRISPR has also been used for widespread screening of functional lncRNAs in cancer. These screens use a library of paired guide RNAs (pgRNAs) to direct the CRISPR/Cas system to cause large deletions that ensure disrupted function in hundreds or thousands of candidate target lncRNAs, which can be subsequently validated by targeted knockdown or activation. A change in cellular growth compared to controls with no deletion can then elucidate function. For example, a CRISPR pgRNA library was used to cause multiple large-scale deletions, targeting approximately 700 human lncRNAs in a liver cancer cell line. Of these, 51 lncRNAs were identified that affected tumour growth, nine of which were further validated using targeted knockdowns and activation [120].

While CRISPR technologies have been successfully used to study ncRNAs in cancers and cancer cell lines, applying these methods to transmissible cancers may present significant challenges. Transmissible cancers can have different genome structure from their host organism. DFTD has a karyotype distinct from the Tasmanian Devil karyotype, with chromosome fragmentation and large-scale rearrangements [28]. CTVT also has a significantly different genomic structure than the normal dog genome, although the copy number appears to be similar [13]. This would present significant challenges for any sequencing technology used to study transmissible cancers with respect to mapping sequence data, a de novo tumour genome assembly would be required. In the absence of an assembly, designing guild RNAs for CRIPSR/Cas experiments would be difficult. Problems of this nature also apply to BTNs, which also have no genome assemblies, and the added complexity of cross species infection.

Many ncRNAs are involved in cancer and perturbing expression of these loci helps to elucidate their roles in tumorigenesis. Transmissible cancers should be no exception, with shared immune system evasion strategies apparent in these rare tumours. However, there is currently no published research on the ncRNAs involved in the biology of transmissible cancers, presenting a significant knowledge gap in this area. Further studies on regulatory mechanisms involving ncRNAs in these tumours will help in understanding the ecology and evolution of transmissible cancers more broadly, and give insight into the tumourigenesis of these unique cancers.

## 4. The Non-Coding RNA Biology of Transmissible Cancers

The non-coding RNA biology of transmissible cancers is poorly understood, so provides a novel avenue of molecular research. Mechanistic studies examining phenotypes produced following CRISPR overexpression or knockdown of target ncRNAs would offer new insights into the functioning of all transmissible cancers. The potential benefits for bivalve aquaculture are clear, which is a global industry that was worth approximately $24 billion (USD) in 2015 [54].

CRISPR/Cas screens have been performed to identify the functional roles of lncRNAs in human-derived cancer cell lines [120,121], but have not been carried out for transmissible cancers. Although no stable cell lines currently exist for BTN, there are cell lines readily available for DFTD and CTVT [122]. These provide the possibility for CRISPR/Cas screens to start disentangling the lncRNA biology that contributes to the proliferation and transmissibility of tumours.

Telomerase is responsible for telomere extensions, and contributes to a cell line becoming immortalised [123]. Importantly, lncRNAs such as telomerase RNA (TER) and telomere repeat-containing RNA (TERRA) are known to be involved in regulating telomerase expression in species from animals to plants to fungi [124,125]. Telomerase is upregulated in both CTVT and DFTD, so is presumably critical for telomere maintenance over long time periods of continual transmission [2,20,32], and could be an important shared characteristic of transmissible cancers, as it is in most non-transmissible tumours [126]. The upregulation of telomerase in DFTD is the result of increased expression of the catalytic subunit of telomerase, TERT [32]. The lncRNA TER is known to interact with TERT to maintain telomeres [125]. Additionally, the lncRNA TERRA specifically recruits TERT to short telomeres to lengthen them and avoid apoptosis [124]. The roles of TER and TERRA in regulating telomerase in transmissible cancers are currently unknown. Their knockdown or activation in both CTVT and DFTD could elucidate a common function in transmissible tumours.

Endogenous retroviruses (ERVs) may also play a role in transmissible tumour biology. Retroviruses have invaded genomes throughout mammal evolution, leaving behind copies of themselves in host genomes that have become ERVs [127]. ERVs can lead to genome instability and cancer. There are a few ncRNAs that have originated from ERV sequences [128]. These can be hard to identify, as RNA-seq mapping can place several of these sequences within the same genomic loci when they may be spaced throughout the genome. Long read sequencing technologies can overcome these shortcomings and identify ncRNAs originating from ERVs [129,130]. As marsupial genomes have the highest prevalence of transposable elements amongst vertebrates [131,132], it is possible that there are ERV-derived ncRNA targets in DFTD for CRISPR knockdown studies. Such studies could be extended to all transmissible tumours, to determine if ERVs are broadly important in transmissible cancer.

It is interesting to note the parallels between transmissible cancers and parasites in terms of host interactions and lifecycle. The ability of BTNs to survive outside their host in the marine environment can be compared to the lifecycle of parasites—growing within the host then spreading into the environment for reproduction (in the case of parasites). Parasites and transmissible cancers also share some aspects of host evasion, downregulating the host organism’s immune response to increase survival [133]. Perhaps there are ncRNAs with common function between the two that enhance their ability to spread, as well as suppress their host immune system.

The MHC is a family of genes integral to vertebrate adaptive immunity, facilitating self/non-self-recognition via polymorphic cell surface markers (MHC-I molecules) [11,134]. Individualised cell surface MHC-I expression allows an animal to recognise and attack foreign material (i.e., transmissible cancer cells). It is therefore unsurprising that both CTVT and DFTD tumour cells have evolved to downregulate MHC. In CTVT, DLA class I and II genes (canine equivalent to MHC-I and -II) are downregulated, which helps it evade the immune system [2,16]. MHC-I is not presented on the surface of DFT1 cells, so it escapes the immune system [33]. In DFT2 MHC-I molecules are presented on tumour cells, so it must currently have a different strategy for escaping host immune responses [35]. It has been proposed that DFT2 may eventually evolve to downregulate MHC-I expression, imitating the evolutionary path of DFT1 [34,35].

In humans, ncRNAs are involved in MHC regulation [135]. An RNA called lncRNA inducing MHC-I and immunogenicity of tumour (LIMIT) was activated (using CRISPR) [136] and found to indirectly cause MHC-I transcription, so was claimed to have a cancer immunogenic function. Similar approaches could be used to examine the function of ncRNAs suspected to be involved in MHC regulation in dog and Tasmanian devil. While MHC expression is not relevant to BTN (bivalves do not have a MHC), ncRNA-dependent MHC regulation could be a key aspect of vertebrate transmissible cancers.

Tasmanian devils naturally lose T cell receptor beta chain (TCRB) diversity as they age beyond the first year of life [137]. TCRB diversity in important for responding to pathogens and cancers, with higher TCRB diversity associated with improved outcomes [137]. DFTD infection causes even lower TCRB diversity in host devils [137]. TCRBs are a component of αβ T cells, which are the most numerous T cell type and require MHC cell surface markers to identify their targets [138]. A smaller proportion of T cells are γδ T cells, which do not require these MHC markers [138]. It is currently unknown how DFTD impacts γδ T cells, but they are of particular interest because they can recognise cellular stress markers, such as those that result from tumorigenesis, independently of MHC haplotype [138]. This is important because reduced MHC cell surface expression in DFT1 [33] leaves αβ T cells unable to recognise the invading tumour, and γδ T cells as one of few defences.

LncRNAs are vital to the regulation of immune cell development; some examples of function are differentiation of dendritic cells (lnc-DC) [139], regulation of the interferon-γ region following infection (NeST) [140] and activation of γδ T cells (TANCR) [141]. Given that γδ T cells have the potential to combat tumours without cell surface MHC-I molecules, γδ T cell expression levels may be of importance in immune evasion in all transmissible cancers. Therefore, understanding the lncRNA biology of DFTD could shed light on its transmission and invasiveness. RNA-seq data from normal Tasmanian devil neuroectoderm and both DFTDs would identify targets of interest for knockdown (CRISPRi) and activation (CRISPRa) assays. Changes in cell phenotype, or expression of a nearby gene through *cis* interaction, would indicate the functional importance of a given lncRNA and be paramount to the first understanding of transmissible tumour RNA biology.

The bivalve immune system is distinct from the mammalian immune system shared by domestic dogs and Tasmanian devils. While bivalves do have a comprehensive innate immune system with self/non-self-recognition capabilities, they do not possess the adaptive immune system seen in vertebrates [142]. It is conceivable that the simpler bivalve immune system permits easier spread of BTN between hosts.

Unfortunately, there is a knowledge gap when it comes to lncRNA cancer biology in bivalves. This lack of information emphasises the need to investigate lncRNAs in BTN, so we can begin to understand their roles and importance. However, there has been increased study of miRNAs in bivalves, showing that they function in stress responses and the immune system [143,144]. To build a balanced understanding of ncRNA biology in BTN we need to advance our knowledge of other bivalve ncRNAs.

Of particular concern is the potential for BTN to impact bivalve aquaculture, which would have severe economic consequences (see Section 1.3). Aquaculture of oysters has already been unstable in virtually every location around the world, usually resulting in the introduction of non-native oyster species following disease or overfishing [145]. Considering that ocean temperatures are predicted to increase, the likelihood of disseminated neoplasia outbreaks may also increase [53]. Therefore, we need a greater understanding of transmissible cancer biology to address these future problems. Improving our understanding of ncRNA biology in transmissible tumours affecting a variety of organisms (dogs, Tasmanian devils and bivalves) will contribute to our overall knowledge of contagious cancer and may identify similarities that could be targeted to treat such an infection. Improved knowledge of transmissible cancer ncRNA biology may benefit the pet industry, as CTVT has the potential to cause economic losses should it spread amongst commercial breeding animals.

## 5. Conclusions

RNA biology presents many avenues of exploration into transmissible tumour biology. Given their ability to alter and regulate gene expression, ncRNAs have potential to provide new insights into invasiveness, transmissibility, and immune evasion. Specific pathways of interest to perturb include ncRNAs involved with telomerase, MHC, T cells and ERVs. This initial mechanistic understanding could be useful in developing potential therapeutics for existing and yet-to-emerge transmissible cancer lineages. A basic understanding of ncRNA biology in any transmissible tumour will better equip us to combat the potential of BTN outbreaks in bivalve aquaculture. This information would also apply to CTVT outbreaks in commercial dog breeding and DFTDs in wild Tasmanian devils. In summary, ncRNAs have the potential to play important roles in transmissible cancer biology, and using CRISPR/Cas to investigate their function would improve on our limited knowledge, likely benefiting the aquaculture, pet and conservation industries.

## Data Availability

Not applicable.

## References

[1] Metzger M.J., Goff S.P. (2016). A Sixth Modality of Infectious Disease: Contagious Cancer from Devils to Clams and Beyond. PLoS Pathog..

[2] Dujon A., Gatenby R.A., Bramwell G., MacDonald N., Dohrmann E., Raven N., Schultz A., Hamede R., Gérard A.-L., Giraudeau M. (2020). Transmissible Cancers in an Evolutionary Perspective. iScience.

[3] Ujvari B., Gatenby R.A., Thomas F. (2016). The evolutionary ecology of transmissible cancers. Infect. Genet. Evol..

[4] Tolar J., Neglia J. (2003). Transplacental and Other Routes of Cancer Transmission Between Individuals. J. Pediatr. Hematol..

[5] Muehlenbachs A., Bhatnagar J., Agudelo C.A., Hidron A., Eberhard M.L., Mathison B.A., Frace M.A., Ito A., Metcalfe M.G., Rollin D.C. (2015). Malignant Transformation of Hymenolepis nana in a Human Host. N. Engl. J. Med..

[6] Gärtner H.-V., Seidl C., Luckenbach C., Schumm G., Seifried E., Ritter H., Bültmann B. (1996). Genetic Analysis of a Sarcoma Accidentally Transplanted from a Patient to a Surgeon. N. Engl. J. Med..

[7] Scanlon E.F., Hawkins R.A., Fox W.W., Smith W.S. (1965). Fatal homotransplanted melanoma: A case report. Cancer.

[8] Chapman J.R., Webster A.C., Wong G. (2013). Cancer in the transplant recipient. Cold Spring Harb. Perspect. Med..

[9] Gandhi M.J., Strong D.M. (2007). Donor derived malignancy following transplantation: A review. Cell Tissue Bank..

[10] Engels E.A., Pfeiffer R.M., Fraumeni J.F., Kasiske B.L., Israni A.K., Snyder J.J., Wolfe R.A., Goodrich N.P., Bayakly A.R., Clarke C.A. (2011). Spectrum of Cancer Risk Among US Solid Organ Transplant Recipients. JAMA.

[11] Dujon A.M., Bramwell G., Roche B., Thomas F., Ujvari B. (2021). Transmissible cancers in mammals and bivalves: How many examples are there? Predictions indicate widespread occurrence. Bioessays.

[12] Das U., Das A.K. (2000). Review of Canine Transmissible Venereal Sarcoma. Vet. Res. Commun..

[13] Murchison E.P., Wedge D.C., Alexandrov L.B., Fu B., Martincorena I., Ning Z., Tubio J.M.C., Werner E.I., Allen J., De Nardi A.B. (2014). Transmissible Dog Cancer Genome Reveals the Origin and History of an Ancient Cell Lineage. Science.

[14] Strakova A., Murchison E.P. (2014). The changing global distribution and prevalence of canine transmissible venereal tumour. BMC Vet. Res..

[15] Baez-Ortega A., Gori K., Strakova A., Allen J.L., Allum K.M., Bansse-Issa L., Bhutia T.N., Bisson J.L., Briceño C., Domracheva A.C. (2019). Somatic evolution and global expansion of an ancient transmissible cancer lineage. Science.

[16] Murgia C., Pritchard J.K., Kim S.Y., Fassati A., Weiss R.A. (2006). Clonal Origin and Evolution of a Transmissible Cancer. Cell.

[17] Rebbeck C.A., Thomas R., Breen M., Leroi A.M., Burt A. (2009). Origins and evolution of a transmissible cancer. Evolution.

[18] Liao K.-W., Lin Z.-Y., Pao H.-N., Kam S.-Y., Wang F.-I., Chu R.-M. (2003). Identification of canine transmissible venereal tumor cells using in situ polymerase chain reaction and the stable sequence of the long interspersed nuclear element. J. Vet. Diagn. Investig..

[19] Katzir N., Rechavi G., Cohen J., Unger T., Simoni F., Segal S., Cohen D., Givol D. (1985). “Retroposon” insertion into the cellular oncogene c-myc in canine transmissible venereal tumor. Proc. Natl. Acad. Sci. USA.

[20] Chu R.M., Lin C.Y., Liu C.C., Yang S.Y., Hsiao Y.W., Hung S.W., Pao H.N., Liao K.W. (2002). Proliferation characteristics of canine transmissible venereal tumor. Anticancer Res..

[21] Hawkins C., Baars C., Hesterman H., Hocking G., Jones M., Lazenby B., Mann D., Mooney N., Pemberton D., Pyecroft S. (2006). Emerging disease and population decline of an island endemic, the Tasmanian devil Sarcophilus harrisii. Biol. Conserv..

[22] Pearse A.M., Swift K. (2006). Allograft theory: Transmission of devil facial-tumour disease. Nature.

[23] Pyecroft S.B., Pearse A.-M., Loh R., Swift K., Belov K., Fox N., Noonan E., Hayes D., Hyatt A., Wang L. (2007). Towards a Case Definition for Devil Facial Tumour Disease: What Is It?. EcoHealth.

[24] Pye R., Pemberton D., Tovar C., Tubio J., Dun K., Fox S., Darby J., Hayes D., Knowles G.W., Kreiss A. (2015). A second transmissible cancer in Tasmanian devils. Proc. Natl. Acad. Sci. USA.

[25] Murchison E.P., Tovar C., Hsu A., Bender H.S., Kheradpour P., Rebbeck C.A., Obendorf D., Conlan C., Bahlo M., Blizzard C.A. (2010). The Tasmanian Devil Transcriptome Reveals Schwann Cell Origins of a Clonally Transmissible Cancer. Science.

[26] Stammnitz M., Coorens T., Gori K., Hayes D., Fu B., Wang J., Martin-Herranz D.E., Alexandrov L.B., Baez-Ortega A., Barthorpe S. (2018). The Origins and Vulnerabilities of Two Transmissible Cancers in Tasmanian Devils. Cancer Cell.

[27] Murchison E.P., Schulz-Trieglaff O.B., Ning Z., Alexandrov L.B., Bauer M.J., Fu B., Hims M., Ding Z., Ivakhno S., Stewart C. (2012). Genome Sequencing and Analysis of the Tasmanian Devil and Its Transmissible Cancer. Cell.

[28] Deakin J.E., Bender H., Pearse A.-M., Rens W., O’Brien P.C.M., Ferguson-Smith M.A., Cheng Y., Morris K., Taylor R., Stuart A. (2012). Genomic Restructuring in the Tasmanian Devil Facial Tumour: Chromosome Painting and Gene Mapping Provide Clues to Evolution of a Transmissible Tumour. PLoS Genet..

[29] McCallum H., Tompkins D.M., Jones M., Lachish S., Marvanek S., Lazenby B., Hocking G., Wiersma J., Hawkins C.E. (2007). Distribution and Impacts of Tasmanian Devil Facial Tumor Disease. EcoHealth.

[30] Lazenby B.T., Tobler M.W., Brown W.E., Hawkins C.E., Hocking G.J., Hume F., Huxtable S., Iles P., Jones M., Lawrence C. (2018). Density trends and demographic signals uncover the long-term impact of transmissible cancer in Tasmanian devils. J. Appl. Ecol..

[31] Moroishi T., Hansen C., Guan K.-L. (2015). The emerging roles of YAP and TAZ in cancer. Nat. Rev. Cancer.

[32] Ujvari B., Pearse A.-M., Taylor R., Pyecroft S., Flanagan C., Gombert S., Papenfuss A.T., Madsen T., Belov K. (2012). Telomere Dynamics and Homeostasis in a Transmissible Cancer. PLoS ONE.

[33] Siddle H.V., Kreiss A., Tovar C., Yuen C.K., Cheng Y., Belov K., Swift K., Pearse A.-M., Hamede R., Jones M. (2013). Reversible epigenetic down-regulation of MHC molecules by devil facial tumour disease illustrates immune escape by a contagious cancer. Proc. Natl. Acad. Sci. USA.

[34] Ong C.E.B., Lyons A.B., Woods G.M., Flies A.S. (2019). Inducible IFN-γ Expression for MHC-I Upregulation in Devil Facial Tumor Cells. Front. Immunol..

[35] Caldwell A., Coleby R., Tovar C., Stammnitz M.R., Kwon Y.M., Owen R.S., Tringides M., Murchison E.P., Skjødt K., Thomas G.J. (2018). The newly-arisen Devil facial tumour disease 2 (DFT2) reveals a mechanism for the emergence of a contagious cancer. eLife.

[36] Epstein B., Jones M., Hamede R., Hendricks S., McCallum H., Murchison E.P., Schönfeld B., Wiench C., Hohenlohe P., Storfer A. (2016). Rapid evolutionary response to a transmissible cancer in Tasmanian devils. Nat. Commun..

[37] Wright B., Willet C.E., Hamede R., Jones M., Belov K., Wade C.M. (2017). Variants in the host genome may inhibit tumour growth in devil facial tumours: Evidence from genome-wide association. Sci. Rep..

[38] Ujvari B., Hamede R., Peck S., Pemberton D., Jones M., Belov K., Madsen T. (2016). Immunoglubolin dynamics and cancer prevalence in *Tasmanian devils* (*Sarcophilus harrisii*). Sci. Rep..

[39] Farley C.A. (1969). Sarcomatoid Proliferative Disease in a Wild Population of Blue Mussels (*Mytilus edulis*). J. Natl. Cancer Inst..

[40] Farley C.A. (1969). Probable neoplastic disease of the hematopoietic system in oysters, *Crassostrea virginica* and *Crassostrea gigas*. Natl. Cancer Inst. Monogr. Ser..

[41] Carballal M.J., Barber B.J., Iglesias D., Villalba A. (2015). Neoplastic diseases of marine bivalves. J. Invertebr. Pathol..

[42] da Silva P.M., Farias N.D., Queiroga F.R., Hégaret H., Soudant P. (2018). Disseminated neoplasia in cultured *Crassostrea gasar* oysters from northeast Brazil. J. Invertebr. Pathol..

[43] Metzger M.J., Reinisch C., Sherry J., Goff S.P. (2015). Horizontal Transmission of Clonal Cancer Cells Causes Leukemia in Soft-Shell Clams. Cell.

[44] Metzger M.J., Villalba A., Carballal M.J., Iglesias D., Sherry J., Reinisch C., Muttray A.F., Baldwin S.A., Goff S.P. (2016). Widespread transmission of independent cancer lineages within multiple bivalve species. Nature.

[45] Yonemitsu A.M., Giersch R.M., Polo-Prieto M., Hammel M., Simon A., Cremonte F., Avilés F.T., Merino-Véliz N., Burioli E.A., Muttray A.F. (2019). A single clonal lineage of transmissible cancer identified in two marine mussel species in South America and Europe. eLife.

[46] Murchison E.P. (2016). Cancer: Transmissible tumours under the sea. Nature.

[47] Skazina M., Odintsova N., Maiorova M., Ivanova A., Väinölä R., Strelkov P. (2021). First description of a widespread Mytilus trossulus-derived bivalve transmissible cancer lineage in *M. trossulus* itself. Sci. Rep..

[48] Aguilera F. (2017). Neoplasia in Mollusks: What Does it Tell us about Cancer in Humans?—A Review. J. Genet. Disord..

[49] Walker C.W., Van Beneden R.J., Muttray A.F., Böttger S.A., Kelley M.L., Tucker A.E., Thomas W.K. (2011). p53 Superfamily Proteins in Marine Bivalve Cancer and Stress Biology. Adv. Mar. Biol..

[50] Sunila I., Farley C. (1989). Environmental limits for survival of sarcoma cells from the soft-shell clam *Mya arenaria*. Dis. Aquat. Org..

[51] Green T., Jones B.J., Adlard R.D., Barnes A. (2008). Parasites, pathological conditions and mortality in QX-resistant and wild-caught Sydney rock oysters, *Saccostrea glomerata*. Aquaculture.

[52] Alderman D., Van Banning P., Perez-Colomer A. (1977). Two European oyster (*Ostrea edulis*) mortalities associated with an abnormal haemocytic condition. Aquaculture.

[53] Bramwell G., Schultz A.G., Sherman C.D., Giraudeau M., Thomas F., Ujvari B., Dujon A.M. (2021). A review of the potential effects of climate change on disseminated neoplasia with an emphasis on efficient detection in marine bivalve populations. Sci. Total Environ..

[54] Olivier A.V.D.S., Jones L., Le Vay L., Christie M., Wilson J., Malham S.K. (2018). A global review of the ecosystem services provided by bivalve aquaculture. Rev. Aquac..

[55] Ohno S. (1972). So much “junk” DNA in our genome. Brookhaven Symp. Boil..

[56] Ohno S., Yomo T. (1991). The grammatical rule for all DNA: Junk and coding sequences. Electrophoresis.

[57] Waterston R.H., Lindblad-Toh K., Birney E., Rogers J., Abril J.F., Agarwal P., Agarwala R., Ainscough R., Alexandersson M., Mouse Genome Sequencing Consortium (2002). Initial sequencing and comparative analysis of the mouse genome. Nature.

[58] Goodstadt L., Ponting C.P. (2006). Phylogenetic Reconstruction of Orthology, Paralogy, and Conserved Synteny for Dog and Human. PLoS Comput. Biol..

[59] International Chicken Genome Sequencing Consortium (2004). Sequence and comparative analysis of the chicken genome provide unique perspectives on vertebrate evolution. Nature.

[60] Mattick J.S., Makunin I.V. (2006). Non-coding RNA. Hum. Mol. Genet..

[61] Guttman M., Amit I., Garber M., French C., Lin M.F., Feldser D., Huarte M., Zuk O., Carey B.W., Cassady J.P. (2009). Chromatin signature reveals over a thousand highly conserved large non-coding RNAs in mammals. Nature.

[62] Mortazavi A., Williams A.B., McCue K., Schaeffer L., Wold B.J. (2008). Mapping and quantifying mammalian transcriptomes by RNA-Seq. Nat. Methods.

[63] Nagalakshmi U., Wang Z., Waern K., Shou C., Raha D., Gerstein M., Snyder M. (2008). The Transcriptional Landscape of the Yeast Genome Defined by RNA Sequencing. Science.

[64] Lister R., O’Malley R.C., Tonti-Filippini J., Gregory B.D., Berry C.C., Millar A.H., Ecker J.R. (2008). Highly Integrated Single-Base Resolution Maps of the Epigenome in Arabidopsis. Cell.

[65] Palade G.E. (1955). A small particulate component of the cytoplasm. J. Cell Biol..

[66] Hoagland M.B., Stephenson M.L., Scott J.F., Hecht L.I., Zamecnik P.C. (1958). A soluble ribonucleic acid intermediate in protein synthesis. J. Biol. Chem..

[67] Brockdorff N., Ashworth A., Kay G.F., McCabe V.M., Norris D.P., Cooper P.J., Swift S., Rastan S. (1992). The product of the mouse Xist gene is a 15 kb inactive X-specific transcript containing no conserved ORF and located in the nucleus. Cell.

[68] Brown C., Ballabio A., Rupert J.L., LaFreniere R.G., Grompe M., Tonlorenzi R., Willard H.F. (1991). A gene from the region of the human X inactivation centre is expressed exclusively from the inactive X chromosome. Nature.

[69] Brannan C.I., Dees E.C., Ingram R.S., Tilghman S.M. (1990). The product of the H19 gene may function as an RNA. Mol. Cell. Biol..

[70] Zhang Y., Tycko B. (1992). Monoallelic expression of the human H19 gene. Nat. Genet..

[71] Carninci P., Kasukawa T., Katayama S., Gough J., Frith M.C., Maeda N., Oyama R., Ravasi T., Lenhard B., Wells C. (2005). The transcriptional landscape of the mammalian genome. Science.

[72] Katayama S., Tomaru Y., Kasukawa T., Waki K., Nakanishi M., Nakamura M., Nishida H., Yap C.C., Suzuki M., Kawai J. (2005). Antisense transcription in the mammalian transcriptome. Science.

[73] Morris K., Mattick J. (2014). The rise of regulatory RNA. Nat. Rev. Genet..

[74] Cheng J., Kapranov P., Drenkow J., Dike S., Brubaker S., Patel S., Long J., Stern D., Tammana H., Helt G. (2005). Transcriptional Maps of 10 Human Chromosomes at 5-Nucleotide Resolution. Science.

[75] Frith M., Pheasant M., Mattick J. (2005). Genomics: The amazing complexity of the human transcriptome. Eur. J. Hum. Genet..

[76] Derrien T., Johnson R., Bussotti G., Tanzer A., Djebali S., Tilgner H., Guernec G., Martin D., Merkel A., Knowles D.G. (2012). The GENCODE v7 catalog of human long noncoding RNAs: Analysis of their gene structure, evolution, and expression. Genome Res..

[77] Rahman L., Bliskovski V., Kaye F.J., Zajac-Kaye M. (2003). Evolutionary conservation of a 2-kb intronic sequence flanking a tissue-specific alternative exon in the PTBP2 gene. Genomics.

[78] Kim T.-K., Hemberg M., Gray J.M. (2015). Enhancer RNAs: A Class of Long Noncoding RNAs Synthesized at Enhancers: Figure 1. Cold Spring Harb. Perspect. Biol..

[79] Pei B., Sisu C., Frankish A., Howald C., Habegger L., Mu X.J., Harte R., Balasubramanian S., Tanzer A., Diekhans M. (2012). The GENCODE pseudogene resource. Genome Biol..

[80] Gibb E.A., Brown C.J., Lam W.L. (2011). The functional role of long non-coding RNA in human carcinomas. Mol. Cancer.

[81] Gutschner T., Diederichs S. (2012). The hallmarks of cancer: A long non-coding RNA point of view. RNA Biol..

[82] Prensner J., Chinnaiyan A.M. (2011). The Emergence of lncRNAs in Cancer Biology. Cancer Discov..

[83] Rinn J.L., Chang H.Y. (2012). Genome Regulation by Long Noncoding RNAs. Annu. Rev. Biochem..

[84] Saayman S., Ackley A., Turner A.-M., Famiglietti M., Bosque A., Clemson M., Planelles V., Morris K. (2014). An HIV-Encoded Antisense Long Noncoding RNA Epigenetically Regulates Viral Transcription. Mol. Ther..

[85] Guil S., Esteller M. (2012). Cis-acting noncoding RNAs: Friends and foes. Nat. Struct. Mol. Biol..

[86] Khalil A.M., Guttman M., Huarte M., Garber M., Raj A., Morales D.R., Thomas K., Presser A., Bernstein B.E., van Oudenaarden A. (2009). Many human large intergenic noncoding RNAs associate with chromatin-modifying complexes and affect gene expression. Proc. Natl. Acad. Sci. USA.

[87] Tsai M.-C., Manor O., Wan Y., Mosammaparast N., Wang J.K., Lan F., Shi Y., Segal E., Chang H.Y. (2010). Long Noncoding RNA as Modular Scaffold of Histone Modification Complexes. Science.

[88] Lee J., Davidow L.S., Warshawsky D. (1999). Tsix, a gene antisense to Xist at the X-inactivation centre. Nat. Genet..

[89] Smilinich N.J., Day C.D., Fitzpatrick G.V., Caldwell G.M., Lossie A.C., Cooper P., Smallwood A.C., Joyce J.A., Schofield P.N., Reik W. (1999). A maternally methylated CpG island in KvLQT1 is associated with an antisense paternal transcript and loss of imprinting in Beckwith-Wiedemann syndrome. Proc. Natl. Acad. Sci. USA.

[90] Wutz A., Smrzka O.W., Schweifer N., Schellander K., Wagner E.F., Barlow D.P. (1997). Imprinted expression of the Igf2r gene depends on an intronic CpG island. Nat. Cell Biol..

[91] Chen J., Sun M., Kent W.J., Huang X., Xie H., Wang W., Zhou G., Shi R.Z., Rowley J.D. (2004). Over 20% of human transcripts might form sense-antisense pairs. Nucleic Acids Res..

[92] Faghihi M.A., Zhang M., Huang J., Modarresi F., Van der Brug M.P., Nalls M.A., Cookson M.R., St-Laurent G., Wahlestedt C. (2010). Evidence for natural antisense transcript-mediated inhibition of microRNA function. Genome Biol..

[93] Mahmoudi S., Henriksson S., Corcoran M., Méndez-Vidal C., Wiman K., Farnebo M. (2009). Wrap53, a Natural p53 Antisense Transcript Required for p53 Induction upon DNA Damage. Mol. Cell.

[94] Tufarelli C., Sloane-Stanley J.A., Garrick D., Sharpe J.A., Ayyub H., Wood W.G., Higgs D.R. (2003). Transcription of antisense RNA leading to gene silencing and methylation as a novel cause of human genetic disease. Nat. Genet..

[95] Morris K., Santoso S., Turner A.-M., Pastori C., Hawkins P.G. (2008). Bidirectional Transcription Directs Both Transcriptional Gene Activation and Suppression in Human Cells. PLoS Genet..

[96] Beltran M., Puig I., Peña C., García J.M., Alvarez A.B., Peña R., Bonilla F., de Herreros A.G. (2008). A natural antisense transcript regulates Zeb2/Sip1 gene expression during Snail1-induced epithelial-mesenchymal transition. Genes Dev..

[97] Choi P., Jordan C.D., Mendez E., Houck J., Yueh B., Farwell D.G., Futran N., Chen C. (2008). Examination of Oral Cancer Biomarkers by Tissue Microarray Analysis. Arch. Otolaryngol. Head Neck Surg..

[98] Taniue K., Akimitsu N. (2021). The Functions and Unique Features of LncRNAs in Cancer Development and Tumorigenesis. Int. J. Mol. Sci..

[99] Rinn J., Kertesz M., Wang J., Squazzo S.L., Xu X., Brugmann S.A., Goodnough L.H., Helms J.A., Farnham P., Segal E. (2007). Functional Demarcation of Active and Silent Chromatin Domains in Human HOX Loci by Noncoding RNAs. Cell.

[100] Wang W.-T., Han C., Sun Y.-M., Chen T.-Q., Chen Y.-Q. (2019). Noncoding RNAs in cancer therapy resistance and targeted drug development. J. Hematol. Oncol..

[101] Lister N., Shevchenko G., Walshe J.L., Groen J., Johnsson P., Vidarsdóttir L., Grander D., Ataide S.F., Morris K.V. (2017). The molecular dynamics of long noncoding RNA control of transcription in PTEN and its pseudogene. Proc. Natl. Acad. Sci. USA.

[102] Johnsson P., Ackley A., Vidarsdottir L., Lui W.-O., Corcoran M., Grandér D., Morris K. (2013). A pseudogene long-noncoding-RNA network regulates PTEN transcription and translation in human cells. Nat. Struct. Mol. Biol..

[103] Carrieri C., Cimatti L., Biagioli M., Beugnet A., Zucchelli S., Fedele S., Pesce E., Ferrer I., Collavin L., Santoro C. (2012). Long non-coding antisense RNA controls Uchl1 translation through an embedded SINEB2 repeat. Nature.

[104] Garneau J.E., Dupuis M., Villion M., Romero D.A., Barrangou R., Boyaval P., Fremaux C., Horvath P., Magadan A.H., Moineau S. (2010). The CRISPR/Cas bacterial immune system cleaves bacteriophage and plasmid DNA. Nature.

[105] Jinek M., Chylinski K., Fonfara I., Hauer M., Doudna J.A., Charpentier E. (2012). A Programmable Dual-RNA–Guided DNA Endonuclease in Adaptive Bacterial Immunity. Science.

[106] Karvelis T., Gasiunas G., Miksys A., Barrangou R., Horvath P., Siksnys V. (2013). crRNA and tracrRNA guide Cas9-mediated DNA interference in *Streptococcus thermophilus*. RNA Biol..

[107] Hsu P., Scott D.A., Weinstein J., Ran F.A., Konermann S., Agarwala V., Li Y., Fine E., Wu X., Shalem O. (2013). DNA targeting specificity of RNA-guided Cas9 nucleases. Nat. Biotechnol..

[108] Shan Q., Wang Y., Li J., Zhang Y., Chen K., Liang Z., Zhang K., Liu J., Xi J.J., Qiu J.-L. (2013). Targeted genome modification of crop plants using a CRISPR-Cas system. Nat. Biotechnol..

[109] Gilbert L., Larson M.H., Morsut L., Liu Z., Brar G.A., Torres S.E., Stern-Ginossar N., Brandman O., Whitehead E.H., Doudna J.A. (2013). CRISPR-Mediated Modular RNA-Guided Regulation of Transcription in Eukaryotes. Cell.

[110] Qi L.S., Larson M.H., Gilbert L., Doudna J.A., Weissman J.S., Arkin A., Lim W.A. (2013). Repurposing CRISPR as an RNA-Guided Platform for Sequence-Specific Control of Gene Expression. Cell.

[111] Polstein L.R., Perez-Pinera P., Kocak D.D., Vockley C.M., Bledsoe P., Song L., Safi A., Crawford G.E., Reddy T.E., Gersbach C.A. (2015). Genome-wide specificity of DNA binding, gene regulation, and chromatin remodeling by TALE-and CRISPR/Cas9-based transcriptional activators. Genome Res..

[112] McDonald J.I., Celik H., Rois L.E., Fishberger G., Fowler T., Rees R., Kramer A., Martens A., Edwards J.R., Challen G.A. (2016). Reprogrammable CRISPR/Cas9-based system for inducing site-specific DNA methylation. Biol. Open.

[113] Hanahan D., Weinberg R.A. (2000). The hallmarks of cancer. Cell.

[114] Frese K.K., Tuveson D.A. (2007). Maximizing mouse cancer models. Nat. Rev. Cancer.

[115] Sánchez-Rivera F.J., Jacks T. (2015). Applications of the CRISPR–Cas9 system in cancer biology. Nat. Rev. Cancer.

[116] Paralkar V.R., Taborda C.C., Huang P., Yao Y., Kossenkov A.V., Prasad R., Luan J., Davies J., Hughes J.R., Hardison R. (2016). Unlinking An lncRNA from Its Associated cis Element. Mol. Cell.

[117] Yi Z., Yan P., Lu J., Song G., Zhu Y., Li Z., Zhao Y., Shen B., Huang X., Zhu H. (2015). Opposing Roles for the lncRNA Haunt and Its Genomic Locus in Regulating HOXA Gene Activation during Embryonic Stem Cell Differentiation. Cell Stem. Cell.

[118] Rankin C.R., Treger J., Faure-Kumar E., Benhammou J., Anisman-Posner D., Bollinger A.E., Pothoulakis C., Padua D.M. (2019). Overexpressing Long Noncoding RNAs Using Gene-activating CRISPR. J. Vis. Exp..

[119] Ghosh S., Tibbit C., Liu J.L. (2016). Effective knockdown of Drosophila long non-coding RNAs by CRISPR interference. Nucleic Acids Res..

[120] Zhu S., Li W., Liu J., Chen C.-H., Liao Q., Xu P., Xu H., Xiao T., Cao Z., Peng J. (2016). Genome-scale deletion screening of human long non-coding RNAs using a paired-guide RNA CRISPR–Cas9 library. Nat. Biotechnol..

[121] Liu S.J., Horlbeck M.A., Cho S.W., Birk H.S., Malatesta M., He D., Attenello F.J., Villalta J.E., Cho M.Y., Chen Y. (2017). CRISPRi-based genome-scale identification of functional long noncoding RNA loci in human cells. Science.

[122] Zayas Y.R., Molina M.A.F., Guerra R.T., Padilla C.R. (2019). Evaluation of a canine transmissible venereal tumour cell line with tumour immunity capacity but without tumorigenic property. J. Vet. Res..

[123] Collins K., Mitchell J.R. (2002). Telomerase in the human organism. Oncogene.

[124] Oliva-Rico D., Herrera L.A. (2017). Regulated expression of the lncRNA TERRA and its impact on telomere biology. Mech. Ageing Dev..

[125] Nelson A.D.L., Shippen D.E. (2015). Evolution of TERT-interacting lncRNAs: Expanding the regulatory landscape of telomerase. Front. Genet..

[126] Harley C.B. (2008). Telomerase and cancer therapeutics. Nat. Rev. Cancer.

[127] Katzourakis A., Magiorkinis G., Lim A.G., Gupta S., Belshaw R., Gifford R. (2014). Larger Mammalian Body Size Leads to Lower Retroviral Activity. PLoS Pathog..

[128] Zhou B., Qi F., Wu F., Nie H., Song Y., Shao L., Han J., Wu Z., Saiyin H., Wei G. (2019). Endogenous Retrovirus-Derived Long Noncoding RNA Enhances Innate Immune Responses via Derepressing RELA Expression. mBio.

[129] Nilsson M.A. (2015). The devil is in the details: Transposable element analysis of the Tasmanian devil genome. Mob. Genet. Elem..

[130] Gallus S., Hallström B.M., Kumar V., Dodt W.G., Janke A., Schumann G.G., Nilsson M.A. (2015). Evolutionary Histories of Transposable Elements in the Genome of the Largest Living Marsupial Carnivore, the Tasmanian Devil. Mol. Biol. Evol..

[131] Sotero-Caio C.G., Platt R.N., Suh A., Ray D.A. (2017). Evolution and Diversity of Transposable Elements in Vertebrate Genomes. Genome Biol. Evol..

[132] Mikkelsen T.S., Wakefield M.J., Aken B., Amemiya C.T., Chang J.L., Duke S., Garber M., Gentles A.J., Goodstadt L., Heger A. (2007). Genome of the marsupial *Monodelphis domestica* reveals innovation in non-coding sequences. Nature.

[133] Calegari-Silva T.C., Vivarini A.C., Pereira R.M.S., Dias-Teixeira K.L., Rath C.T., Pacheco A.S.S., Silva G.B.L., Pinto C.A.S., Dos Santos J.V., Saliba A.M. (2018). *Leishmania amazonensis* downregulates macrophage iNOS expression via Histone Deacetylase 1 (HDAC1): A novel parasite evasion mechanism. Eur. J. Immunol..

[134] Piertney S.B., Oliver M.K. (2005). The evolutionary ecology of the major histocompatibility complex. Heredity.

[135] Chitnis N.S., Shieh M., Monos D. (2020). Regulatory noncoding RNAs and the major histocompatibility complex. Hum. Immunol..

[136] Li G., Kryczek I., Nam J., Li X., Li S., Li J., Wei S., Grove S., Vatan L., Zhou J. (2021). LIMIT is an immunogenic lncRNA in cancer immunity and immunotherapy. Nature.

[137] Cheng Y., Makara M., Peel E., Fox S., Papenfuss A.T., Belov K. (2019). Tasmanian devils with contagious cancer exhibit a constricted T-cell repertoire diversity. Commun. Biol..

[138] Legut M., Cole D.K., Sewell A.K. (2015). The promise of γδ T cells and the γδ T cell receptor for cancer immunotherapy. Cell. Mol. Immunol..

[139] Wang P., Xue Y., Han Y., Lin L., Wu C., Xu S., Jiang Z., Xu J., Liu Q., Cao X. (2014). The STAT3-Binding Long Noncoding RNA lnc-DC Controls Human Dendritic Cell Differentiation. Science.

[140] Gomez J.A., Wapinski O.L., Yang Y.W., Bureau J.F., Gopinath S., Monack D.M., Chang H.Y., Brahic M., Kirkegaard K. (2013). The NeST long ncRNA controls microbial susceptibility and epigenetic activation of the interferon-gamma locus. Cell.

[141] Yang C., Feng T., Lin F., Gong T., Yang S., Tao Y., Li H. (2020). Long noncoding RNA TANCR promotes γδ T cells activation by regulating TRAIL expression in cis. Cell Biosci..

[142] Allam B., Raftos D. (2015). Immune responses to infectious diseases in bivalves. J. Invertebr. Pathol..

[143] Abo-Al-Ela H.G., Faggio C. (2021). MicroRNA-mediated stress response in bivalve species. Ecotoxicol. Environ. Saf..

[144] Rosani U., Bortoletto E., Bai C.-M., Novoa B., Figueras A., Venier P., Fromm B. (2021). Digging into bivalve miRNAomes: Between conservation and innovation. Philos. Trans. R. Soc. B Biol. Sci..

[145] Botta R., Asche F., Borsum J.S., Camp E.V. (2020). A review of global oyster aquaculture production and consumption. Mar. Policy.

